# An Oxidase-Based Electrochemical Fluidic Sensor with High-Sensitivity and Low-Interference by On-Chip Oxygen Manipulation

**DOI:** 10.3390/s120708955

**Published:** 2012-06-29

**Authors:** Nitin Radhakrishnan, Jongwon Park, Chang-Soo Kim

**Affiliations:** 1 Department of Electrical & Computer Engineering, Missouri University of Science and Technology, 301 W. 16th St., Rolla, MO 65409, USA; E-Mails: niradhak@cisco.com, (N.R.); jpark3@kiu.ac.kr (J.P.); 2 Department of Biological Sciences, Missouri University of Science and Technology, 301 W. 16th St., Rolla, MO 65409, USA

**Keywords:** bubble, electrolysis, oxygen, hydrogen, biosensor, oxidase

## Abstract

Utilizing a simple fluidic structure, we demonstrate the improved performance of oxidase-based enzymatic biosensors. Electrolysis of water is utilized to generate bubbles to manipulate the oxygen microenvironment close to the biosensor in a fluidic channel. For the proper enzyme reactions to occur, a simple mechanical procedure of manipulating bubbles was developed to maximize the oxygen level while minimizing the pH change after electrolysis. The sensors show improved sensitivities based on the oxygen dependency of enzyme reaction. In addition, this oxygen-rich operation minimizes the ratio of electrochemical interference signal by ascorbic acid during sensor operation (*i.e.*, amperometric detection of hydrogen peroxide). Although creatinine sensors have been used as the model system in this study, this method is applicable to many other biosensors that can use oxidase enzymes (e.g., glucose, alcohol, phenol, *etc.*) to implement a viable component for in-line fluidic sensor systems.

## 1. Introduction

Oxidase enzymes are one of the most common biorecognition agents being utilized in electrochemical enzymatic biosensors. As shown in Reaction (1), the amount of hydrogen peroxide generated is proportional to that of the substrate (*i.e.*, target analyte) to allow the estimation of the substrate concentration:

(1) $$\text{Substrate}+{\text{H}}_{2}\text{O}+{\text{O}}_{2}\to \text{By‐product}+{\text{H}}_{2}{\text{O}}_{2}\;(\text{with oxidase enzyme})$$

Mostly, the detection of hydrogen peroxide is amperometrically done by the working electrode of sensor that is positively-biased with respect to its reference electrode. Reaction (1), however, implies that the sensor signal depends on the availability of oxygen that is a co-factor of reaction. Consequently, one challenging problem of oxidase biosensors is the signal fluctuation caused by the background oxygen environment (*i.e.*, dissolved oxygen content in the sample solution) during the enzymatic reaction [1–8].

While many approaches have been focused on overcoming this problem [1–8], we worked on developing a method to take advantage of this oxygen dependency to obtain special functionalities of dissolved oxygen and oxidase-based sensors [9,10]. By performing electrolysis of water, a pair of oxygen and hydrogen bubbles is generated at the electrolysis electrodes as follows:

(2) $$2{\text{H}}_{2}\text{O}\to 4{\text{H}}^{+}+4{\text{e}}^{-}+{\text{O}}_{2}\;(\text{anode})$$

(3) $$4{\text{H}}_{2}\text{O}+4{\text{e}}^{-}\to 4{\text{OH}}^{-}+2{\text{H}}_{2}\;(\text{cathode})$$

The oxygen bubble provides oxygen-saturated environment to the sensor located in proximity to the anode, which increases the sensitivity since the reaction is not limited by the oxygen supply. Furthermore, since the analyte-selective signal is “amplified” by a supply of excess oxygen, it is expected that the ratio of any interference signal by undesirable electrochemical reactions to the total signal can be minimized. On the other hand, the hydrogen bubble depletes oxygen near the sensor to mimic the analyte-free sample since the reaction cannot be completed without oxygen, thus providing a possibility to conduct a one-point (zero-value baseline) calibration procedure. In our previous attempt with glucose and lactate sensors [10], the generated bubble made contact directly with the biosensor placed in a fluidic channel in such a way that the active sensing area (*i.e.*, enzyme layer) was completely surrounded by the bubble. As a consequence this configuration isolated the enzyme layer from the bulk solution by the surrounding bubble. This may prevent the diffusion of reaction species between the enzyme matrix and the bulk solution, thus causing the reaction by-products to accumulate within the enzyme layer. In addition, the water electrolysis inevitably accompanies a pH change as in Reactions (2) and (3), which may change enzyme activity. Therefore the pH change near the sensor should be minimized during the bubble generation and manipulation.

In this report, we present an improved fluidic device structure to circumvent any possible artifact aforementioned. The new method involved shifting remotely-generated bubbles which are then stopped by a bubble stopper near the sensor without contacting the active sensing area. The profiles of oxygen and pH inside the fluidic channel were analyzed to ensure the saturation or depletion of oxygen at the sensor location with minimal pH change. A creatinine sensor with a tri-enzyme matrix was used as a model system to verify the effectiveness of new method. The creatinine level is a very important clinical parameter in estimating renal, muscular, and thyroid functions and can be detected electrochemically [11]. A tri-enzyme matrix membrane employing three enzymes, namely creatinine amidohydrolase (CA), creatine amidinohydrolase (CI) and sarcosine oxidase (SO) were used [12]. The creatinine sensor performance was investigated by the chronoamperometric method at the optimum fluidic conditions.

## 2. Experimental Section

### 2.1. Chip Design and Fabrication

**Figure 1.**
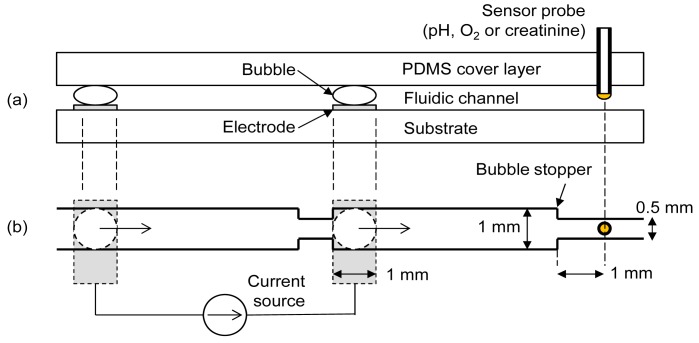
(**a**) Side view of fluidic device (150 μm channel height); (**b**) Top view of the channel pattern for final distance of 1 mm between bubble and sensor (1 mm nominal width; 0.5 mm bubble stopper). An oxygen (hydrogen) bubbles are generated at the right-side electrode with a positive (negative) current and shifted towards bubble stopper. The exposed electrode area within the channel is 1 mm × 1 mm.

To achieve the maximum degree of oxygen manipulation with minimum pH artifact, two important parameters needed to be optimized; the final distance between bubble and sensor following shifting of bubble and the current density for electrolytic bubble generation. Experiments were conducted using a commercial pH sensor and an oxygen sensor to determine the optimum values of these parameters. The fluidic chip contained a cover layer, an electrode substrate and a sensor probe as in Figure 1(a). The cover layer was made of polydimethylsiloxane (PDMS) containing the fluidic channel patterns. Separate needle-type sensors (pH, oxygen or creatinine) were interchangeably inserted into the elastic PDMS cover layer for different measurements. A pair of oxygen and hydrogen bubbles was generated by a pair of platinum electrodes on the substrate. A bubble stopper as shown in Figure 1(b) was placed at the required distances so that the final distances between the sensor and bubble stood at 5 mm, 3 mm, and 1 mm, respectively, after bubble shifting. Figure 1 shows the case of 1 mm design. The right-side electrode is separated by 5 mm from the sensor probe, implying that there is no bubble shifting procedure for the case of the 5 mm design. Depending on the polarity of current, either oxygen or hydrogen bubble was generated at the right-side electrode. This right-side bubble controlled the oxygen microenvironment near the sensor. The used bubbles were easily removed by a simple fluidic manipulation.

A silicon wafer with silicon nitride layer coating was used as the electrode substrate. A platinum/titanium thin film (100 nm/20 nm) was deposited by e-beam evaporation and patterned by lift-off technique to define the electrodes. The PDMS cover layer was prepared by a molding technique with a negative template patterned by a photoresist [9]. The photoresist used was of negative tone, high aspect ratio, and epoxy-based photopolymer (SU-8 2050, MicroChem). A PDMS channel height of about 150 μm was necessary to locate the sensor distal tip at the middle of the channel by inserting it manually. To achieve this, triple coating of the photoresist was needed. A silicon wafer was first subjected through a cleaning process involving acetone, methyl alcohol and deionized (DI) water. The first layer of photoresist was spin-coated at 1,000 rpm on the silicon wafer. This was subjected to soft-baking times of 5 min and 30 min at temperatures of 65 °C and 95 °C, respectively. The second layer of SU-8 was spin-coated at 1,000 rpm on the existing soft-baked first layer. Soft-baking times of 7 min and 45 min at temperatures of 65 °C and 105 °C, respectively, were chosen. Finally the third layer was spin-coated and baked as the same condition with the second layer. The channel pattern on the photomask was transferred to the SU-8 by exposing it with UV rays. The wafer was subjected to post-baking times of 5 min and 15 min at temperatures of 65 °C and 95 °C, respectively. Finally the unexposed part of SU-8 was removed by putting it in a developer solution for 15 min. The silicon wafer with channel patterns was used as a negative template for the molding process of the PDMS (Sylgard184, Dow Corning) cover layer. The curing of about one cm thick PDMS was done for 24 h at room temperature in a vacuum desiccator.

### 2.2. Creatinine Sensor Preparation

The three enzymes sequentially convert creatinine to creatine and finally to sarcosine and hydrogen peroxide as shown in Reactions (4) to (6) [12]:

(4) $$\text{Creatinine}+{\text{H}}_{2}\text{O}\to \text{Creatine}\;(\text{with creatinine amidohydrolase})$$

(5) $$\text{Creatine}+{\text{H}}_{2}\text{O}\to \text{Sarcosine}+\text{Urea}\;(\text{with creatine amidinohydrolase})$$

(6) $$\text{Sarcosine}+{\text{H}}_{2}\text{O}+{\text{O}}_{2}\to \text{Glycine}+\text{Formaldehyde}+{\text{H}}_{2}{\text{O}}_{2}\;(\text{with sarcosine oxidase})$$

For the preparation of tri-enzyme layer, glutaraldehyde, creatinine amidohydrolase (CA), creatine amidinohydrolase (CI), sarcosine oxidase (SO) and bovine serum albumin (BSA) were used. All were obtained from Sigma (Sigma-Aldrich Co.). Phosphate buffer (PB, pH 7.4, 10 mM) was prepared by mixing NaH_2_PO_4_ and KH_2_PO_4_. Creatinine and ascorbic acid were also obtained from Sigma. A needle-type, two-electrode amperometric creatinine microsensor consisted of a teflon-coated platinum (Pt) wire (A-M Systems, Inc.) with a diameter of 0.008″ as working electrode and a chloridized silver (Ag) tube (GoodFellow Inc.) with an inner diameter of 0.3 mm as reference electrode, respectively. The Pt wire with a teflon cladding was inserted into the Ag tube. The teflon layer served as an insulation layer between the two electrodes. Triethoxysilane (1 wt%) was deposited on the distal tip of this coaxial electrode and was cured for 30 min at 80 °C. Then the tri-enzyme mixture (CA, CI, SO) and BSA were mixed in the weight ratio of 1.0:5.0. Finally the mixture was dissolved in PB in the weight ratio of 1.00:5.67. The enzyme solution was transferred to the exposed distal tip of Pt electrode and dried for 30 min at room temperature. Then glutaraldehyde (5 wt%) was transferred to the sensor tip and cured for another 30 min at room temperature for crosslinking of the enzyme layer. The final sensor cross-section is shown in Figure 1(a). The whole sensor assembly was immersed in PB and then kept in refrigerator for 24 h before use. The creatinine solutions were prepared by mixing the appropriate quantities of creatinine in PB.

### 2.3. Measurement

The PDMS cover layer was aligned on the substrate so that the electrode pairs were exposed within the channel. The sealing between them was done by simply pressing the cover layer against the substrate. A small hole was made in the PDMS cover layer to insert the needle-type sensor (either pH, oxygen or creatinine). The oxygen sensor used was a commercial fiber optic microprobe (OxyMicro, WPI Inc.) with a fiber diameter of 125 μm. Two-point calibration of the oxygen sensor was done in a beaker filled with DI water by purging oxygen gas or nitrogen gas for 15 min before use. The pH sensor (AMANI, Innovative Instruments Inc.) has tip diameter of 0.45 mm. For the pH sensor, three-point calibration was done with three calibration solutions of pH 4, pH 7 and pH 10 before use.

To generate bubbles, an electrochemical instrument (FAS1, Gamry Instruments) was operated in galvanostatic mode (*i.e.*, controlled current). Current densities of 3.5, 5.5, 7.5, and 12.0 mA/cm^2^ were chosen to generate bubbles. The bubble was generated for 2 min by applying each current density and was then shifted toward the sensor by applying pressure from the left-side of the channel in Figure 1. It was almost instantaneously stopped by the bubble stopper. The pH and oxygen readings were analyzed to find the optimum distance between the bubble and sensor and the current density for electrolysis. After determining the optimum values, creatinine measurements were done by biasing the sensor working electrode (Pt) at 0.8 V with respect to its reference electrode (Ag/AgCl) using the chronoamperometric mode (*i.e.*, controlled voltage) of the electrochemical instrument. The bubble was generated for 2 min at optimized current density and the chronoamperometric measurement was started at 30 s after shifting the bubble. An electrochemical interference test was conducted with a creatinine solution containing ascorbic acid.

## 3. Results and Discussion

### 3.1. pH Analysis

**Figure 2.**
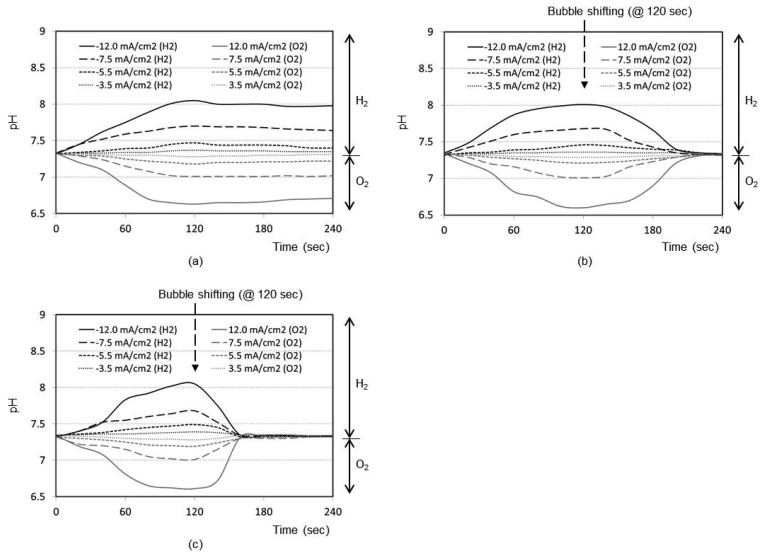
Responses of pH sensor during bubble generation (2 min) and shifting towards bubble stopper. Sample pH at the sensor location increases during the hydrogen generation, while it decreases during the oxygen generation, respectively. (**a**) Final distance of 5 mm (*i.e.*, no shifting); (**b**) Final distance of 3 mm; (**c**) Final distance of 1 mm.

Figure 2(a) shows the time responses of a pH sensor for a final distance between the bubble and pH sensor of 5 mm (*i.e.*, no shifting). After the bubble generation with the maximum current density of 12.0 mA/cm^2^ for 2 min, the pH change at the pH sensor location increases up to 8.1 with the hydrogen bubble and decreases down to 6.7 with the oxygen bubble, respectively. Without shifting the bubbles, the changed pH recovers back to the original pH of sample solution very slowly. Figures 2(b,c) shows the same kind of curve sets obtained for final distance of 3 mm and 1 mm, respectively, after bubble shifting. As expected, the general trend of the respective curves was the same as that of 5 mm design. However, the recovery to original pH after the bubble shifting is much faster. It is considered that the perturbation of solution expedites the pH buffering process as the bubble shifts. From Figure 2, it is evident that closer the bubble moves to the sensor (1 mm), quicker is the recovery of pH to its original value (about 30 s).

Real samples (e.g., tissue fluid), however, include electrochemically active species that may affect the current efficiency for bubble generation and lead to the generation of undesirable redox by-products. Therefore, to minimize this possibility a special electrode with a low-overpotential for water electrolysis should be used as the electrolysis electrode for bubble generation. One candidate, Pt-black, is known to exhibit a low overpotential and has an excellent process compatibility for this type of devices by electroplating [13]. Therefore, the future work will employ the Pt-black electrodes.

### 3.2. Oxygen Analysis

**Figure 3.**
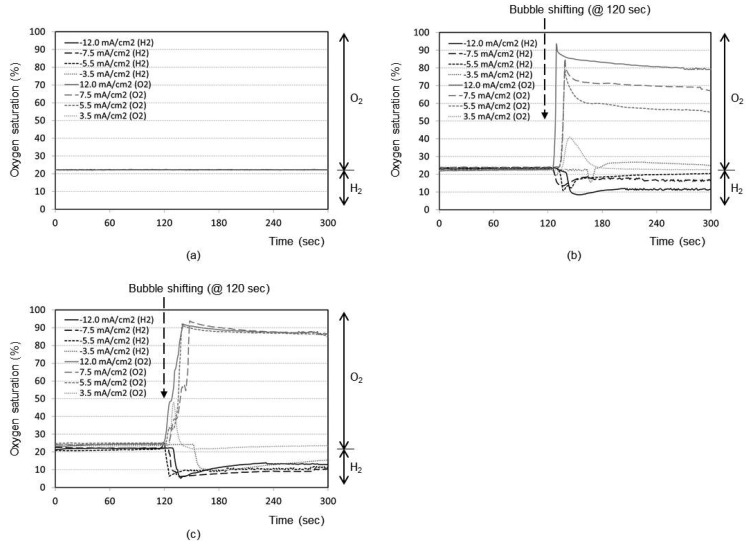
Responses of oxygen sensor during bubble generation (2 min) and shifting towards bubble stopper. Sample oxygen at the sensor location decreases when the hydrogen bubble arrives, while it increases when the oxygen bubble arrives, respectively. (**a**) Final distance of 5 mm (*i.e.*, no shifting); (**b**) Final distance of 3 mm; (**c**) Final distance of 1 mm.

Figure 3(a) represents the time responses of oxygen saturation percentage for final distance between oxygen sensor and bubble as 5 mm. The plots show that the oxygen saturation remains around 21% (*i.e.*, air saturation) for all current densities when no shifting of bubble has taken place after the bubble generation phase. The bubble generated at maximum distance of 5 mm from the sensor is not effective at all to cause any change in oxygen environment near the sensor. It is considered that a significant portion of generated dissolved oxygen nucleates the bubble at the electrode surface rather than migrating into the bulk solution. In addition, the lower diffusion coefficient of oxygen in water (2.42 × 10^−5^ cm^2^/s), than those of proton (9.31 × 10^−5^ cm^2^/s) and hydroxyl ion (5.27 × 10^−5^ cm^2^/s) [14], appears to contribute to this little response compared to that of pH in Figure 2(a).

The time responses are shown in Figure 3(b) for final distance of 3 mm. During the bubble generation phase of 2 min, the oxygen concentration remains almost at 21% for all current densities. For the lowest current density of 3.5 mA/cm^2^ for oxygen, it is seen that the oxygenation state returns to the baseline shortly after the transient change up to 40%. This implies that the small size bubble with a low current density does not induce a significant effect near the sensor. For maximum current density of 12.0 mA/cm^2^, however, the oxygen saturation and depletion reaches approximately 93% and 8%, respectively, which then returns to the baseline very slowly. In Figure 3(c) for the final distance of 1 mm, for all current densities except the lowest one, the respective oxygen saturation and depletion percentages initially stand at around 92% and 7%, respectively. The recovery is, however, slower compared to Figure 3(b), which is advantageous for our application. The lowest current density of 3.5 mA/cm^2^ still remains ineffective. We believe that any adsorbed species on the electrode surface from the previous opposite-polarity electrolysis reaction can be eliminated during the new electrolysis phase of 120 s.

### 3.3. Creatinine Measurements

Many facts were confirmed from the pH and oxygen analyses. As expected, the higher the current density used, the easier to reach the oxygen saturation or depletion states. The closer the bubble moves to the sensor, the higher is the degree of oxygen saturation or depletion. For our application, we need to choose optimum current density and distance to reach the maximum oxygen saturation or depletion state with minimal pH perturbation. From Figures 2 and 3, it can be concluded that the optimum condition is 5.5 mA/cm^2^ with a final distance of 1 mm. Also from Figures 2(c) and 3(c), the best timing to start the chronoamperometric creatinine measurement (*i.e.*, application of step-wise potential to working electrode to result in well-known exponentially decreasing current responses) was determined to be 30 s after bubble shifting when the pH was recovered to its original value while the oxygen change still remains effective. Therefore, all creatinine measurements were done under these conditions.

**Figure 4.**
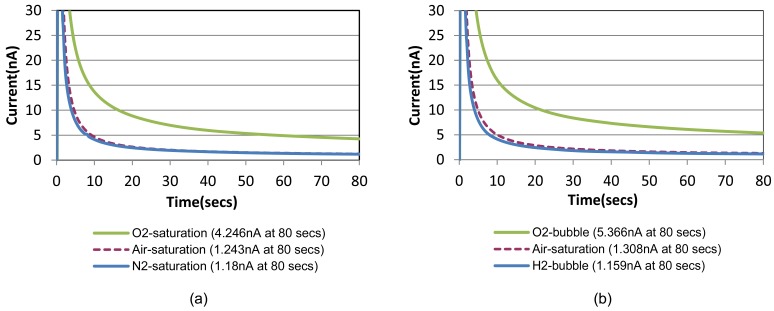
Comparison of chronoamperometric time responses for a creatinine concentration of 10 mg/dL (t = 0 s corresponds to t = 150 s in Figure 3). (**a**) Sensor placed in a beaker filled with a creatinine solution saturated or depleted of oxygen by purging with oxygen or nitrogen cylinder. (**b**) Same sensor mount on the fluidic chip at optimized final distance 1 mm and current density 5.5 mA/cm^2^.

Chronoamperometric responses of the prepared sensor are shown in Figure 4(a) for sample solutions with a fixed creatinine concentration of 10 mg/dL with three different oxygenation states. They are nitrogen-saturated (*i.e.*, oxygen-depleted), air-saturated, and oxygen-saturated states established by purging gases into the sample solution in a beaker. Figure 4(b) shows the responses for the same creatinine concentration with the different oxygenation conditions achieved by the bubble method in the chip. A step-wise 0.8 V potential was applied to the sensor working electrode at t = 0 s that is same as t = 150 s in Figure 3 (*i.e.*, 30 safter the shifting). Figure 4 compares very similar chronoamperometric responses and oxygen dependency from these two measurement approaches using a same sensor. This fact confirms that our simple fluidic chip can effectively serve as a miniaturized oxygen manipulator for our application.

**Figure 5.**
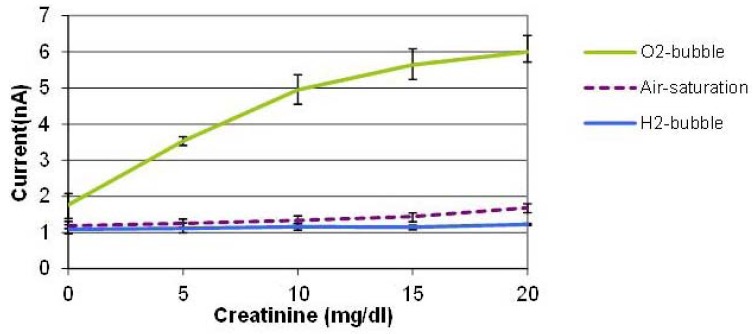
Sensor signal versus creatinine concentration (read at 80 s). The creatinine solutions were saturated or depleted of oxygen by bubbles in fluidic channel at optimized final distance of 1 mm and current density 5.5 mA/cm^2^ (n = 9, error bars = min/max). A higher sensitivity is obtained during the oxygen bubble phase, while the signals are nearly minimal regardless of creatinine concentration during the hydrogen bubble phase.

Figure 5 shows the average values of three measurements conducted with three creatinine sensors prepared by the same procedure on different dates. The error bars indicate the maximum and minimum values. With the oxygen bubble, the sensitivity is dramatically higher than that of air-saturated solution. When an oxygen bubble is generated and shifted, the environment around the sensor is nearly saturated with high oxygen content, so the enzyme reaction is not limited by the oxygen availability in solution which leads to a sensitivity enhancement. It was found that the tri-enzyme membrane loses its activity considerably during storage in a refrigerator for several days. Although it is beyond the scope of this paper, more stable enzyme immobilization method should be developed to be combined with this bubble method to demonstrate robust creatinine sensors. The PDMS material used for the cover layer has high oxygen permeability, which means that it is not the ideal material for this application. We used this material, however, because of its elasticity to puncture a hole and insert the sensor probe in the cover layer easily. Therefore, we expect that the use of a channel material with lower oxygen permeability other than PDMS can improve the device performance.

During the hydrogen bubble phase, on the contrary, the signals were minimal and almost constant regardless of the creatinine concentration, which implies that it is promising to realize on-demand, *in situ*, on-chip self-calibration of zero-value correction procedures. When a hydrogen bubble is generated and shifted towards the location of the creatinine biosensor, the oxygen-depleted environment prevents the enzyme reaction from taking place. This mimics the creatinine-free environment even in the presence of creatinine in sample solutions without the need of bringing the analyte-free control solution through externally tethered modules. This capability is potentially very useful for periodic self-correction of baseline and self-diagnosis of sensor viability whenever it is needed during continuous sensor operation.

**Figure 6.**
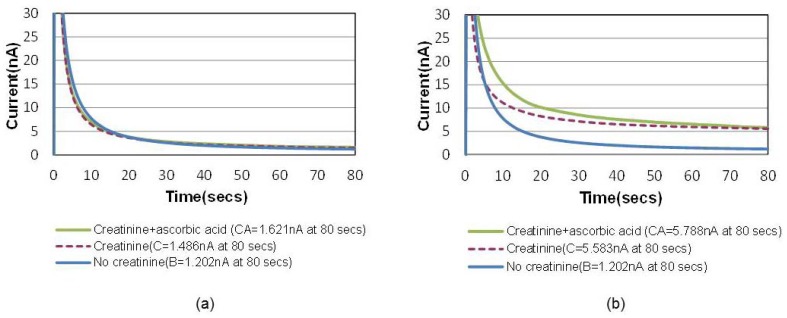
Chronoamperometric responses for a creatinine concentration of 15 mg/dL with and without 1 mg/dL ascorbic acid (t = 0 s corresponds to t = 150 s in Figure 3). (**a**) Responses in a creatinine solution saturated with air; (**b**) Responses in a creatinine solution saturated with an oxygen bubble at optimized final distance of 1 mm and current density 5.5 mA/cm^2^. The ratio of interference signal (*i.e.*, pure creatinine signal/ascorbic noise = [C−B]/[(CA)−C]) is suppressed in (**b**).

A common way to minimize the electrochemical interference of sensors is to introduce a permselective membrane on the active sensing site to screen out the interferents [15]. Another unique advantage of this bubble method is that the “oxygen-amplified” signal effectively suppresses the noise ratio caused by electrochemical interferences, which can possibly eliminate the necessity of the additional membrane. Figure 6(a) shows the chronoamperometric plots of air-saturated 15 mg/dL creatinine solutions with and without 1 mg/dL ascorbic acid, the most common interferent during the electrochemical hydrogen peroxide detection. Figure 6(b) shows the same for an oxygen saturation case created by the bubble in the channel. The electrochemical signal-to-noise ratio (*i.e.*, pure creatinine signal/ascorbic noise = [C−B]/[CA−C] in Figure 6 increased from 2.1 to 21.4 (at 80 s), which is approximately 10 times higher.

## 4. Conclusions/Outlook

A simple, unique method of on-chip manipulation of oxygen environment was explored utilizing the fluidic technique. The “oxygen-amplification” method increased sensitivity, which in turn suppressed the ratio of electrochemical interference in the oxidase-based creatinine sensor signal. The demonstrated system is considered to be a promising platform compatible with in-line fluidic monitoring system. This bubble method is also applicable to other target analytes that can utilize the oxidase enzymes as the recognition agent, including glucose, lactate, acetylcholine, cholesterol, amino acids, alcohol and phenol. Therefore, we expect that this method has a high impact on the monitoring of many substances in medical diagnosis, bioprocess, and environmental areas.

## References

[1] Enfors S. (1981). -O. Oxygen-stabilized enzyme electrode for D-glucose analysis in fermentation broths. Enz. Microb. Technol..

[2] Kusano H. (1989). Glucose enzyme electrode with percutaneous interface which operates independently of dissolved oxygen. Clin. Phys. Physiol. Meas..

[3] Gough D.A., Armour J.C. (1995). Development of the implantable glucose sensor. What are the prospects and why is it taking so long?. Diabetes.

[4] Wang J., Lu F. (1998). Oxygen-rich oxidase enzyme electrodes for operation in oxygen-free solution. J. Am. Chem. Soc..

[5] Wang J., Chen L., Jiang M., Lu F. (1999). Myoglobin-containing carbon-paste enzyme microelectrodes for the biosensing of glucose under oxygen-deficit conditions. Anal. Chem..

[6] Abel P.U., von Woedtke T. (2002). Biosensors for *in vivo* glucose measurement: Can we cross the experimental stage. Biosens. Bioelectron..

[7] Dixon B.M., Lowry J.P., O'Neill R.D. (2002). Characterization *in vitro* and *in vivo* of the oxygen dependence of an enzyme/polymer biosensor for monitoring brain glucose. J. Neurosci. Methods.

[8] Wang J., Li S., Mo J.-W., Porter J., Musameh M.M., Dasgupta P.K. (2002). Oxygen-independent poly(dimethylsiloxane)-based carbon-paste glucose biosensors. Biosens. Bioelectron..

[9] Park J., Kim C.-S., Kim Y. (2005). A simple on-chip self-diagnosis/self-calibration method of oxygen microsensor using electrochemically generated bubbles. Sens. Actuators B Chem..

[10] Park J., Kim C.-S., Choi M. (2006). Oxidase-coupled amperometric glucose and lactate sensors with integrated electrochemical actuation system. IEEE Trans. Instrum. Meas..

[11] Killard A.J., Smyth M.R. (2000). Creatinine biosensors: Principles and designs. Trends Biotechnol..

[12] Tsuchida T., Yoda K. (1983). Multi-enzyme membrane electrodes for determination of creatinine and creatine in serum. Clin. Chem..

[13] Suzuki H., Yoneyama R. (2003). Integrated microfluidic system with electrochemically actuated on-chip pumps and valves. Sens. Actuators B Chem..

[14] Ride D.L. (2007). CRC Handbook of Chemistry and Physics.

[15] Madaras M.B., Buck R.P. (1996). Miniaturized biosensors employing electropolymerized permselective films and their use for creatinine assays in human serum. Anal. Chem..

